# Codon optimization of a gene encoding DNA polymerase from *Pyrococcus furiosus* and its expression in *Escherichia coli*

**DOI:** 10.1186/s43141-023-00605-7

**Published:** 2023-11-21

**Authors:** Isa Nuryana, Fina Amreta Laksmi, Kartika Sari Dewi, Faiz Raihan Akbar, Nurhayati, Rikno Harmoko

**Affiliations:** 1 Research Center for Applied Microbiology, National Research and Innovation Agency, Jalan Raya Bogor Km 46, Cibinong, Bogor 16911 Indonesia; 2 Research Center for Genetic Engineering, National Research and Innovation Agency, Jalan Raya Bogor Km 46, Cibinong, Bogor 16911 Indonesia; 3 Department of Biology, Faculty of Sciences and Mathematics, Universitas Diponegoro, Jalan Prof Soedarto, SH, Kampus UNDIP Tembalang, Semarang, 50275 Indonesia

**Keywords:** Codon optimization, Polymerase activity, Purification, Recombinant enzyme

## Abstract

**Background:**

DNA polymerase is an essential component in PCR assay for DNA synthesis. Improving DNA polymerase with characteristics indispensable for a powerful assay is crucial because it can be used in wide-range applications. Derived from *Pyrococcus furiosus*, *Pfu* DNA polymerase (*Pfu* pol) is one of the excellent polymerases due to its high fidelity. Therefore, we aimed to develop *Pfu* pol from a synthetic gene with codon optimization to increase its protein yield in *Escherichia coli*.

**Results:**

Recombinant *Pfu* pol was successfully expressed and purified with a two-step purification process using nickel affinity chromatography, followed by anion exchange chromatography. Subsequently, the purified *Pfu* pol was confirmed by Western blot analysis, resulting in a molecular weight of approximately 90 kDa. In the final purification process, we successfully obtained a large amount of purified enzyme (26.8 mg/L). Furthermore, the purified *Pfu* pol showed its functionality and efficiency when tested for DNA amplification using the standard PCR.

**Conclusions:**

Overall, a high-level expression of recombinant *Pfu* pol was achieved by employing our approach in the present study. In the future, our findings will be useful for studies on synthesizing recombinant DNA polymerase in *E. coli* expression system.

## Background

DNA polymerases are essential enzymes in molecular biology for directing the synthesis of double-stranded DNA molecules from deoxyribonucleotides. In living organisms, these enzymes play a crucial role in DNA replication as they can polymerize new DNA strands by synthesizing complementary DNA strands according to their template [1, 2]. Furthermore, DNA polymerases maintain genome integrity during DNA replication. The enzymes have wide-range applications for DNA manipulation in vitro such as DNA cloning, sequencing, polymerase chain reaction (PCR), mutagenesis, single-nucleotide polymorphism (SNP) detection, and other purposes [3]. In combination with PCR, DNA polymerases can be used to intensify DNA fragments, named DNA amplification, adapting a similar process of DNA replication in vivo [4, 5].

In the early PCR era, the Klenow (large) fragment of DNA polymerase I from *Escherichia coli* was reported to be used in PCR technology [6]. In addition, a simple yet vigorous method using DNA polymerase from *Thermus aquaticus* (*Taq* pol) was evolved for a denaturation step in PCR assay afterward [7, 8]. Generally, *Taq* pol is the most preferred enzyme in DNA amplification. Due to its thermostability and potent extension ability, the enzyme is extensively developed for PCR kits [9]. However, *Taq* pol has no proofreading ability because of a lack of 3′-5′ exonuclease. Therefore, it is unable to be applied in DNA cloning since high fidelity is crucial and required for the technique [10]. On this basis, DNA polymerase derived from *Pyrococcus furiosus* (*Pfu* pol) is highly desirable. First isolated from the hyperthermophilic archaeon, *Pfu* pol is known to have the lowest error rate of any DNA polymerases. The enzyme exhibits an integrated 3’–5’ exonuclease activity for proofreading and correcting errors during the polymerization [11, 12].

Developing thermostable DNA polymerase with characteristics indispensable for a robust assay becomes the utmost priority. Nowadays, many DNA polymerases have been engineered in *Escherichia coli* expression systems to make them straightforward to purify. Mutant and artificial DNA polymerases have been created to produce recombinant enzymes with improved properties. In addition, specific characteristics such as fidelity, processivity, thermostability, and substrate nucleotide specificity have been further developed for particular purposes, making the enzymes suitable for many kinds of applications and applicable to industries [13, 14]. However, the study related to codon optimization of a gene encoding *Pfu* pol has remained limited. For that reason, we conducted a preliminary study to focus on developing *Pfu* pol from a synthetic gene with codon optimization as a strategy to improve protein yield. Heterologous expression was performed in *Escherichia coli* using an IPTG inducible expression system. The recombinant enzyme was then purified using nickel affinity chromatography and followed by anion exchange chromatography. Finally, the activity of the recombinant enzyme was assessed using a real-time quantitative PCR (qPCR) system and the functionality of the purified enzyme was tested for standard PCR assay.

## Methods

### Plasmid, bacterial strain, and medium

Plasmid pD451-SR harboring *Pfu* DNA polymerase-encoding gene (pD451-SR-Pfupol) was synthetically made by ATUM (US) with codon optimization and used as the expression vector. Meanwhile, *E. coli* DH5α (Invitrogen) was used for plasmid cloning, and *E. coli* BL21 Star (DE3) (Invitrogen) was employed for protein expression. Both strains were cultivated using Luria–Bertani (LB) medium containing per liter: 10 g tryptone, 5 g yeast extract, and 10 g sodium chloride. Bacterial transformant cells were grown in LB medium supplemented with 30 mg L^−1^ kanamycin (LB-kanamycin).

### Design of a synthetic gene encoding *Pfu* DNA polymerase

**Fig. 1:**
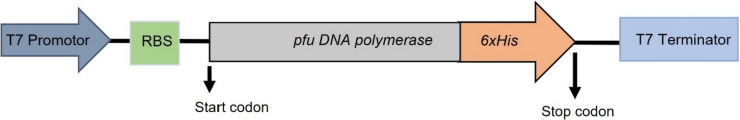
The expression cassette of gene encoding *Pfu* pol

The sequence of the *Pfu* DNA polymerase-encoding gene used in this study was retrieved from the thermophilic archaea *Pyrococcus furiosus* [15]. The gene consists of 2325 base pairs with 775 aa and 90.113 kDa in size. To facilitate the purification process, a 6 × His-tag was fused at the C-terminal end of the gene. The overview of the expression cassette of *Pfu* DNA polymerase gene can be seen in Fig. 1.

The *Pfu* pol-coding sequence was optimized based on the amino acid sequences accessed from UniProtKB-P61875. The *E. coli* B codon usage table from Kazusa codon database was used as a reference [16]. Optimization was performed using Gene Designer and OPTIMIZER software [17, 18]. The codon optimization sequence was then re-evaluated using GenScript rare codon analysis tools and codon adaptation index (CAI) calculator for checking the CAI value, rare codons, GC content, and negative CIS elements. The analysis of alignment was performed using CLUSTALW software for mutation detection [19]. The translation result of the synthetic gene encoding *Pfu* pol was aligned to the amino acid sequence template [20]. Finally, the codon-optimized sequence of *Pfu* pol was synthesized and made by ATUM, Inc.

### Expression and purification of recombinant *Pfu* DNA polymerase in *E. coli*

Preparation of competent cells and bacterial transformation were carried out using a polyethylene glycol (PEG)-mediated transformation method previously described by Chung et al. with modification [21]. Bacterial *E. coli* DH5α transformants were prepared for glycerol stocks and stored in a freezer (− 80 °C) for plasmid storage, while *E. coli* BL21 Star (DE3) transformants were used for colony selection.

Bacterial transformants of *E. coli* BL21 Star (DE3) were pre-cultured into 5 mL LB-kanamycin medium containing 0.4% glucose and incubated with shaking at 37 °C for 16 h. One percent pre-culture was inoculated into LB-kanamycin broth and the culture was incubated with shaking at 37 °C. Once the OD_600_ reached 0.8–1.0, 0.2 mM IPTG was added to induce the expression of the enzyme. For enzyme expression, the bacterial culture was incubated with shaking at 37 °C for 24 h. The cells were then harvested by centrifugation (4 °C, 7871 × g) for 10 min and resuspended in 25 mM Tris–HCl buffer (pH 8.0). The resuspended cells were disrupted by sonication and the cell debris was separated from the supernatant by centrifugation (4 °C, 13,148 × g) for 15 min. The soluble protein-containing supernatant was collected in a new tube, while insoluble protein-containing cell debris was diluted with 25 mM Tris–HCl buffer (pH 8.0) and mixed well using a vortex.

Furthermore, two-step chromatographic purification was applied to purify the crude extract from the supernatant. Firstly, the crude extract was loaded into a HisTrap HP column (Cytiva) on AKTA Prime Plus Liquid Chromatography System (Cytiva). The column was equilibrated using 20 mM sodium phosphate buffer containing 500 mM NaCl and 20 mM imidazole (Buffer A, pH 7.4) at a flow rate of 3 mL min^−1^, while 20 mM sodium phosphate buffer consisting of 500 mM NaCl and a linear inclination of 20–500 mM imidazole (Buffer B, pH 7.4) was flowed through the column at a flow rate of 1 mL min^−1^. Lastly, the target fractions were pooled for continued purification with a HiTrap™ Q HP column (Cytiva) on a similar instrument, which was previously equilibrated using 50 mM Tris–HCl buffer (pH 8.0). The binding fractions in the column were then eluted using 50 mM Tris–HCl buffer (pH 8.0) with a gradient concentration of 0–500 mM NaCl. The target fractions were collected and dialyzed to remove NaCl against 25 mM Tris–HCl buffer (pH 8) at 4 °C overnight. The molecular mass and expression level of protein was analyzed on a 10% gel using sodium dodecyl sulfate–polyacrylamide gel electrophoresis (SDS-PAGE) system according to the previous method [22]. Subsequently, the purity of the protein was evaluated using Western blot, while the concentration of the purified protein was determined using a BCA kit assay (Merck).

### Western blot analysis

Western blotting was performed as previously described [23]. After protein electrophoresis, the protein sample was transferred into a nitrocellulose membrane using a Mini ProteanVR II trans blot unit (Bio-Rad). The sample was then blocked with BSA/TBST at room temperature for 1 h in a shaking incubator. Afterward, washing was carried out twice using 2 × 15 mL TBST for 10 min. HisProbe-HRP solution was used to bind the recombinant *Pfu* pol. Visualization of the recombinant protein was done by adding KPL TMB peroxidase substrate (3,3′,5,5′-tetramethylbenzidine) to the membrane.

### Enzyme activity assay

The enzymatic activity of *Pfu* pol was quantitatively determined using the fluorometric method. The method was assessed using a real-time quantitative PCR (qPCR, Bio-Rad) instrument. Briefly, a qPCR reaction mixture was prepared using EvaEZ™ Fluorometric Polymerase Activity Assay Kit (Biotium) following the manufacturer’s instructions. A commercial DNA polymerase (Toyobo) was used as the known sample. The qPCR program was set as an isothermal reaction at 65 °C for 90 min. Subsequently, the fluorescence readings were obtained each minute and the initial rate of fluorescence change (RFU min^−1^) was achieved during polymerization. Moreover, the slope of the curve was generated by plotting the *X*-axis with time (min) and the *Y*-axis with the relative fluorescence unit (RFU). The activity of the *Pfu* pol sample was obtained by comparing the fluorescence change to the standard curve of the known sample.

The purified *Pfu* pol was tested for its functionality and performance in the standard PCR assay using 1.0 and 1.25 units of the enzyme to amplify a ~ 900-bp region of the inserted gene encoding D-allulose epimerase (DAEase) from plasmid DNA as the template. The polymerase activity was quantitatively determined in the standard DNA polymerase assay conditions in a final volume of 50 μL, consisting of 20 mM Tris–HCl (pH 8.8), 10 mM KCl, 1 mM MgSO_4_, 6 mM (NH_4_)_2_SO_4_, 0.1% Triton X-100, 0.1 mg/mL BSA, 200 μM each of dNTPs, 0.4 μM primer pair, and 1 ng plasmid DNA. The PCR mixture without containing *Pfu* pol was used as a negative control.

## Results

### Design of a synthetic gene encoding *Pfu* DNA polymerase

A codon-optimized gene encoding *Pfu* DNA polymerase was designed according to the *E. coli B* codon usage table. The infrequently used codons in *E. coli* were replaced with preferable ones to optimize the expression of the target enzyme. The codon distribution of the wild-type versus the codon-optimized sequence is shown in Table 1. The table shows that rare codon in *E. coli*, such as AGA and AGG that encodes Arginine, was replaced with preferable codons like CGU and CGC.

**Table 1:** Codon distribution of the wild-type versus the codon-optimized sequence

Amino acid	Codon	Host fraction	*Pfu* DNA polymerase gene	
			Wild-type	Optimized
F	UUU	28.9	14	14
F	UUC	18.8	13	13
L	UUA	17.5	11	2
L	UUG	18.6	7	9
L	CUU	12.7	18	3
L	CUC	14.1	17	1
L	CUA	3.4	13	0
L	CUG	54.9	1	52
I	AUU	33.9	31	31
I	AUC	31.0	7	40
I	AUA	5.0	33	0
M	AUG	37.4	10	10
V	GUU	19.6	23	20
V	GUC	14.3	4	15
V	GUA	10.6	19	1
V	GUG	33.9	6	16
S	UCU	8.5	2	3
S	UCC	8.0	2	4
S	UCA	6.1	7	0
S	UCG	11.4	2	2
P	CCU	5.8	9	2
P	CCC	2.4	6	2
P	CCA	7.4	21	4
P	CCG	24.9	0	30
T	ACU	7.7	14	3
T	ACC	25.2	4	17
T	ACA	6.1	10	0
T	ACG	14.6	3	11
A	GCU	13.8	13	5
A	GCC	25.5	8	8
A	GCA	19.6	19	10
A	GCG	32.6	4	21
Y	UAU	18.6	24	20
Y	UAC	8.5	22	26
*	UAA	1.9	0	1
*	UAG	0.3	1	0
H	CAU	9.3	8	8
H	CAC	7.2	6	12
Q	CAA	13.5	11	7
Q	CAG	24.7	4	8
N	AAU	21.2	9	9
N	AAC	15.9	10	10
K	AAA	29.2	37	43
K	AAG	8.8	45	39
D	GAU	30.0	26	17
D	GAC	15.1	12	21
E	GAA	29.4	50	48
E	GAG	18.0	38	40
C	UGU	4.2	2	2
C	UGC	5.8	2	2
*	UGA	0.8	0	0
W	UGG	12.7	11	11
R	CGU	16.4	1	29
R	CGC	18.8	0	15
R	CGA	2.4	0	0
R	CGG	5.0	0	0
S	AGU	9.0	6	0
S	AGC	14.3	6	16
R	AGA	2.4	28	1
R	AGG	2.1	16	0
G	GGU	24.4	6	32
G	GGC	33.1	8	18
G	GGA	8.2	29	0
G	GGG	14.3	7	0

The difference between the wild-type and the codon-optimized sequences encoding *Pfu* pol, measured with several parameters including CAI, and the percentages of GC content and rare codon as well as negative CIS elements is presented in Table 2. The CAI and GC content increased to 0.8 and 49.5%, respectively, after codon optimization. Conversely, Nc, the percentage of rare codons, and negative CIS elements resulted in decreasing in patterns with respective amounts from 47.4 to 39.9, 17% to 1%, and 5 to 1.

**Table 2:** Comparison of wild-type and codon-optimized sequences using several parameters

Sequences	CAI	GC content (%)	Nc	Rare codon (%)	Negative CIS elements
Wild-type	0.53	39.3	47.4	17	5
Optimized	0.8	49.5	39.9	1	1

### Expression of recombinant *Pfu* DNA polymerase in *E. coli* system

**Fig. 2:**
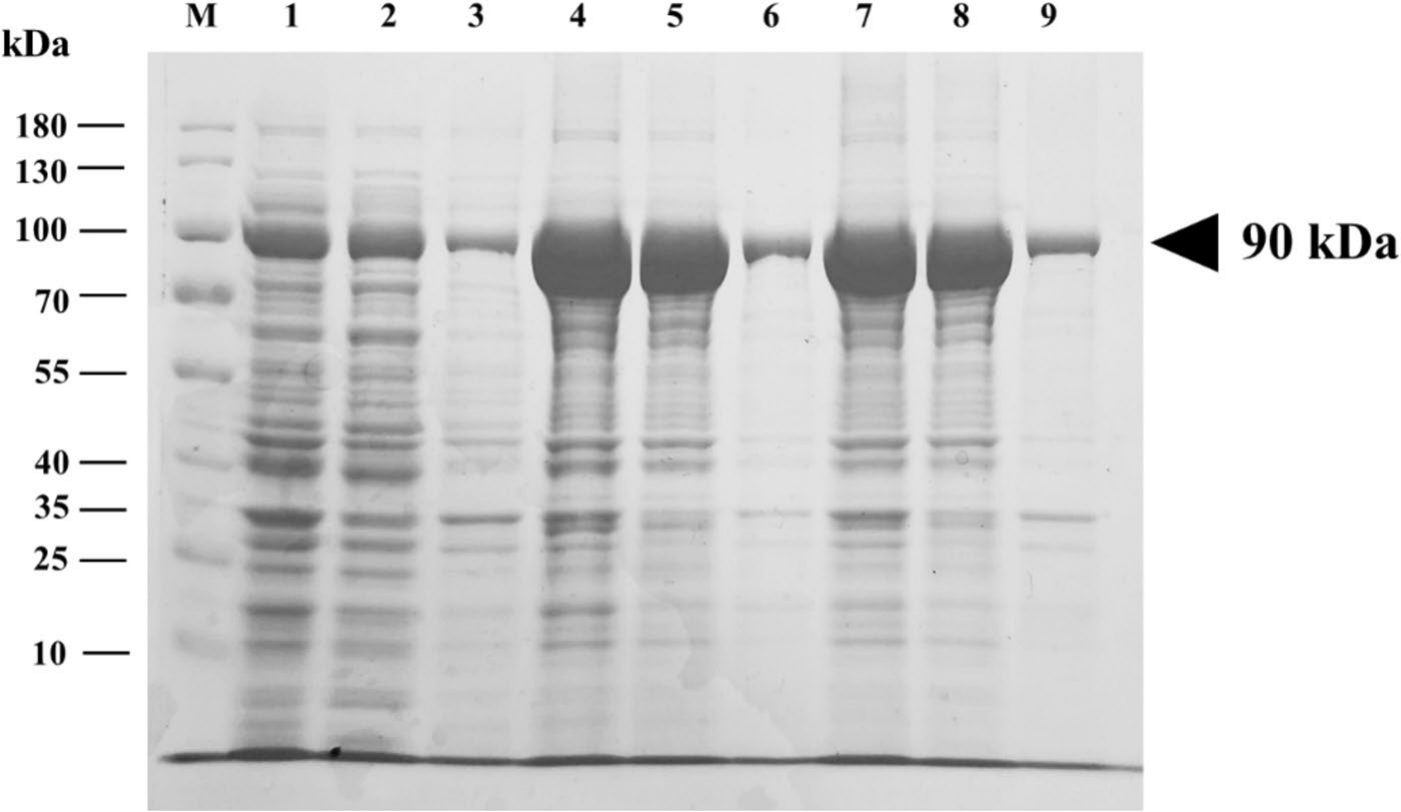
Expression of recombinant protein of *Pfu* pol in *E. coli* BL21 transformants with IPTG induction. The triangle symbol indicates the targeted protein band. Lane M: protein marker; lanes 1, 4, and 7: total fraction; lanes 2, 5, and 8: soluble fraction; lanes 3, 6, and 9: insoluble fraction; lanes 1, 2, and 3: transformant colony number 01 (TC-01); lanes 4, 5, and 6: transformant colony number 02 (TC-02); and lanes 7, 8, and 9: transformant colony number 03 (TC-03)

Three prospective transformants, namely TC-01, TC-02, and TC-03, were screened based on their potential to express the recombinant *Pfu* pol. The total protein expression of the respective transformant, composed of soluble and insoluble fractions, was evaluated using SDS-PAGE analysis. As depicted in Fig. 2, it is obvious that all transformants successfully express recombinant *Pfu* pol in soluble form. The *Pfu* pol-encoding gene with a 775 amino-acid sequence has a molecular weight of roughly 90 kDa. A single colony with a high level of *Pfu* pol expression, TC-02, was selected as the parental colony for further investigation. Subsequently, the colony of TC-02 was employed to express and produce recombinant *Pfu* pol in LB-kanamycin medium induced with 0.2 mM IPTG.

**Fig. 3:**
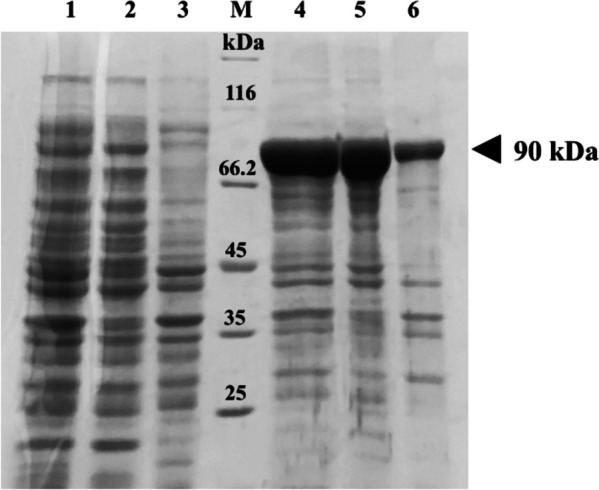
Expression of *Pfu* pol in non-transformant and transformant strains of *E. coli* BL21. The triangle symbol marks the targeted protein band. Lane M: protein marker; lanes 1 and 4: total fraction; lanes 2 and 5: soluble fraction; lanes 3 and 6: insoluble fraction; lanes 1, 2, and 3: non-transformant strain showing no *Pfu* pol expression; and lanes 4, 5, and 6: transformant strain showing overexpressed *Pfu* pol

The comparison of Pfu pol expression in non-transformant and transformant strains of *E. coli* BL21 is depicted in Fig. 3. The high level of *Pfu* pol expression was observed in the transformant’s total fraction whereas no *Pfu* pol expression could be found in the non-transformant’s total fraction. The recombinant protein was confirmed to have a predicted molecular weight of approximately 90 kDa.

### Purification of recombinant *Pfu* DNA polymerase using chromatography

The purity of recombinant *Pfu* pol was achieved after performing a two-step purification process and evaluated using SDS-PAGE and Western blot analyses. Figure 5A shows the total protein obtained from the crude and purified *Pfu* pol after the purification process using two chromatographic columns. All proteins showed the equivalent molecular weight of roughly 90 kDa.

**Fig. 4:**
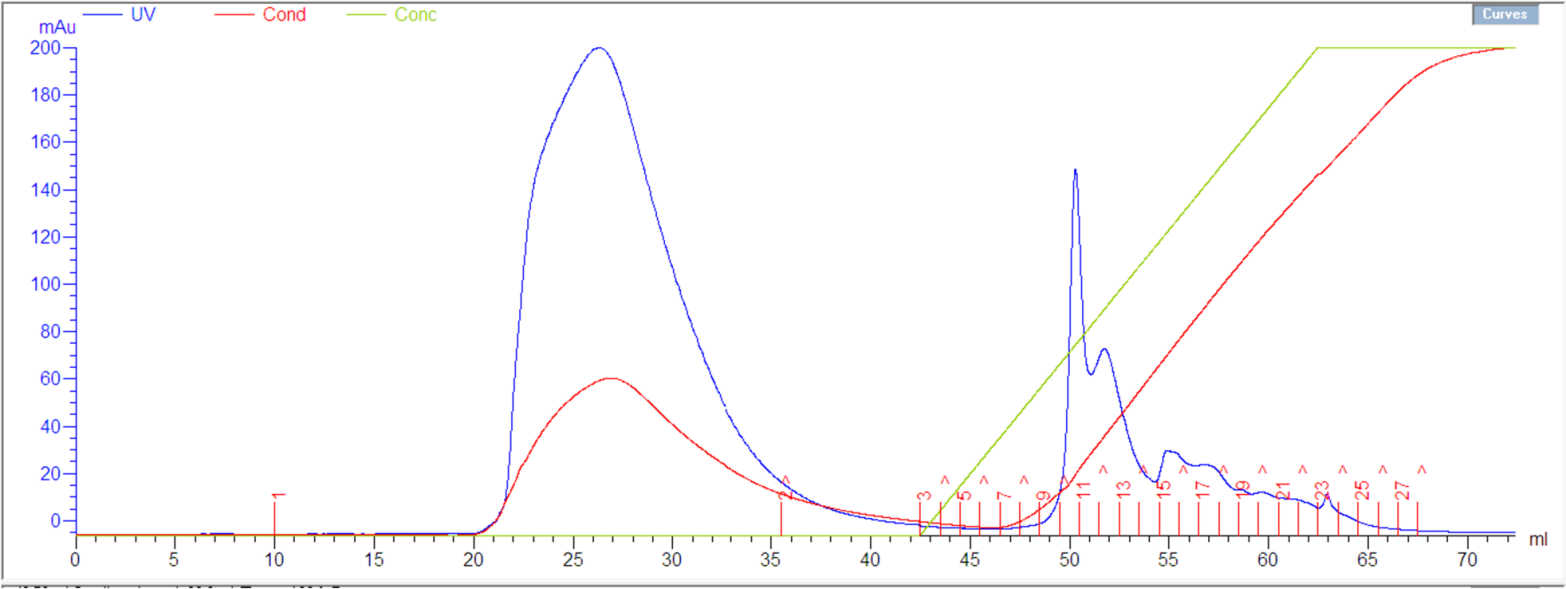
Chromatogram for purification of the recombinant *Pfu* pol using AKTAprime Plus protein purifier system and HiTrap™ Q HP column

Figure 4 shows the chromatogram for purification of the recombinant *Pfu* pol using AKTAprime Plus protein purifier system and HiTrap™ Q HP column. The blue spectrum indicates absorbance at 280 nm and suggests the presence of protein. The broad first peak (between 20 and 35 mL) is the column flowthrough. The narrow second peak (fractions 10 and 11; between 49.5 and 51.5 mL) and the narrow third peak (fractions 12 and 13; between 51.5 and 53.5 mL) are the column eluent. Both peaks indicate the purified *Pfu* pol based on SDS PAGE analysis (figure not shown) and the combination of the enzyme from both peaks was used for activity assay.

**Fig. 5:**
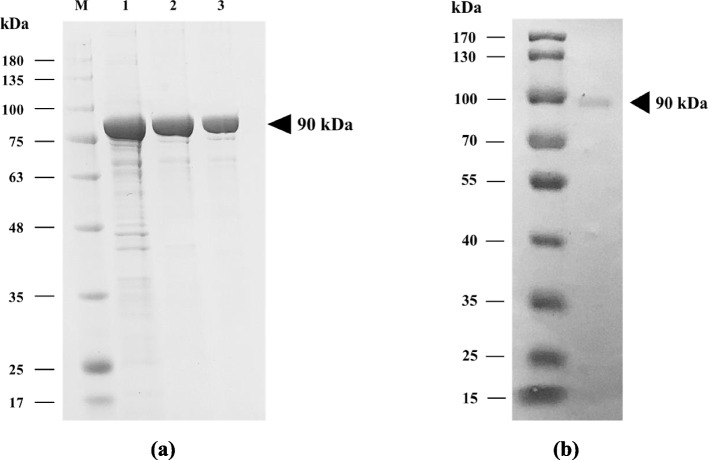
**a** SDS-PAGE analysis. Lane M: protein marker; lane 1: crude protein, lane 2: *Pfu* pol purified using HisTrap™ HP column; and lane 3: *Pfu* pol purified using HiTrap™ Q HP column. **b** Western blot analysis. Lane M: protein marker and lane 1: purified *Pfu* pol. The triangle symbol shows the targeted protein band

Further confirmation of the purified protein and its molecular weight was carried out by Western blot analysis. As described in Fig. 5B, it is confirmed that the identity of purified protein is certainly *Pfu* pol. The molecular weight of a single band obtained remains around 90 kDa, corresponding to the molecular weight predicted from the amino-acid sequence (775 aa). In consequence, the crude and purified *Pfu* pol was processed for polymerase activity determination using a real-time quantitative PCR system.

### The activity and functionality of purified *Pfu* DNA polymerase

Table 3 presents the summary of purification results and relevant details regarding total activity, total protein, and specific activity. Total activity and protein showed downward trends, decreasing from 45,260 U to 19,620 U and 20.09 mg to 1.34 mg in a 50-mL culture, respectively. In contrast, the increased pattern in specific activity of the purified *Pfu* pol (from 2253 U/mg to 14,641 U/mg) is proportional to the purification fold, ranging from 1 to 6.5. In the end product of *Pfu* pol, the yield reached at 43%.

**Table 3:** Comparison of crude and purified *Pfu* pol in a 50-mL culture. The values are mean of triplicate samples ± standard deviation

Purification step	Total activity (U)	Total protein (mg)	Specific activity (U/mg)	Purification (fold)	Yield (%)
Crude	45,260 ± 1530	20.09 ± 1.68	2253	1	100
HisTrap™ HP	21,067 ± 1030	5.02 ± 0.25	4197	1.86	46
HiTrap™ Q HP	19,620 ± 880	1.34 ± 0.08	14,641	6.5	43

**Fig. 6:**
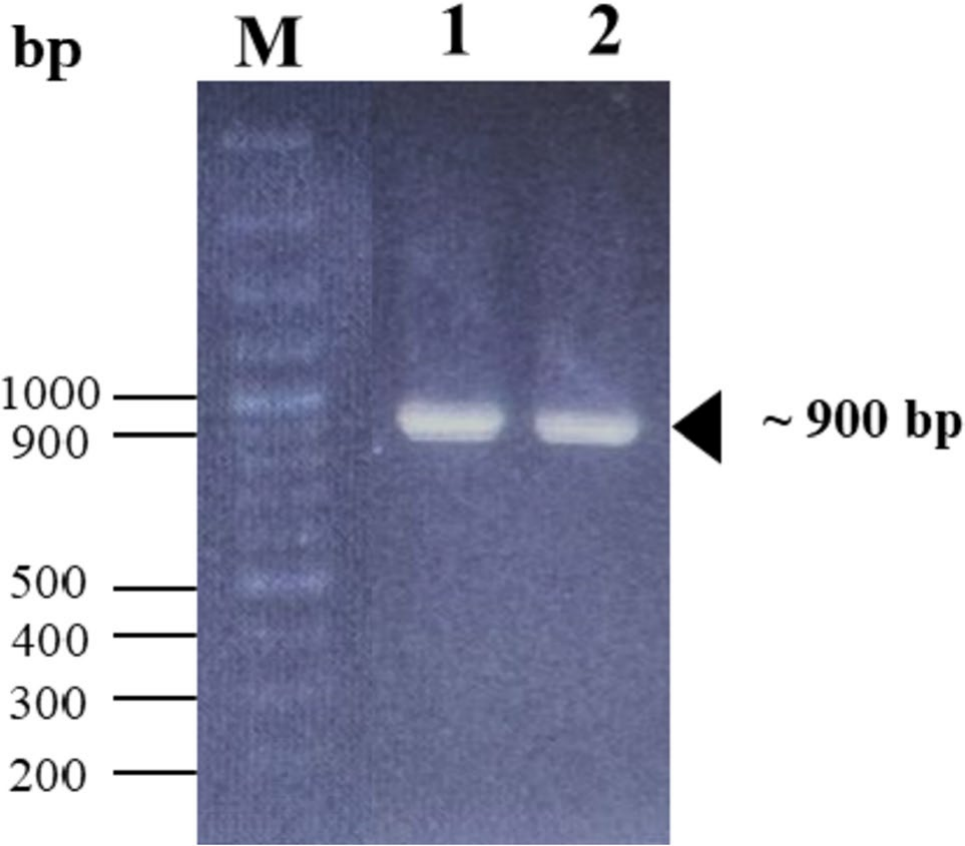
The activity of purified *Pfu* pol in the standard PCR assay. Lane M: 100 bp DNA ladder; lanes 1–2: purified *Pfu* pol of 1 U and 1.25 U, respectively. The triangle symbol shows the targeted DNA band

The functionality of the purified *Pfu* pol was tested for the standard PCR assay using a plasmid DNA as a template to amplify a 900-bp target gene. The result exhibited that the purified *Pfu* pol was fully functional and was able to amplify the target gene, generating a single band with the correct size of roughly 900 base pairs in length. In this study, around 1 and 1.25 units of purified *Pfu* pol were tested and applied for PCR assay. As presented in Fig. 6, all samples show a similar amplification efficiency.

## Discussion

In the present study, codon optimization was designed according to the *E. coli* codon usage, aiming to increase the expression level of the recombinant enzyme. The original sequence from *Pyrococcus furiosus* was modified in order to match with *E. coli* codon usage preference. The strategy for codon optimization began by substituting some codons with synonymous ones that encode the same amino acids for *E. coli*. The principle of this strategy is that the use of frequent codons for protein synthesis in a particular host resulted in a high level of protein expression based on previous studies [24, 25]. Genes with frequent codons are highly desirable for *E. coli* to translate its codons into protein. The presence of rare codons in *E. coli* leads to the low rate of protein translation, thus replacing the original DNA sequence with an optimized one is essential to increase the translation efficiency, which resulted in improved protein production with native conformation and high stability [26, 27]. The use of the synthetic gene allows the replacement of synonymous codons in order to achieve optimal codons. Our previous study has reported that by optimizing codon and culture conditions, the production of recombinant reverse transcriptase could be successfully improved [28]. Additionally, other studies have also revealed that codon substitution showed a significant impact on gene expression level and protein folding [29, 30]. Thus, we attempted to increase the codon frequency of *E. coli* to obtain the high yield of *Pfu* pol.

To understand the positive correlation between codon optimization and recombinant *Pfu* pol expression, several parameters need to be measured such as the CAI and the percentages of GC content and rare codon as well as negative CIS elements. The CAI is the main index that is generally used to predict the level of gene expression, suggesting the extent to which the coding sequence describes the usage of codons in an organism. In the present study, the CAI of the codon-optimized sequence obtained was 0.8. Theoretically, CAI of 0.8–1 is considered to be good and ideal for heterologous expression in the host of interest. The lower the number, the higher probability that the gene can be expressed poorly [31]. The GC content was increased from 39.3 to 49.5%, getting closer to the GC content of the *E. coli* expression host (51.06%). Moreover, to eliminate rare codons, there are also some parameters existing with notable impacts on protein expression, such as the GC content, cleavages, and restriction sites, as well as RNA secondary structure [32]. The percentage of low-frequency codons of codon-optimized sequence based on the *E. coli* codon usage is about 1%, showing much lower than the wild-type one. The efficiency of recombinant protein expression can be significantly improved by lowering the number of low-frequency codons as it can avoid ribosome stalling during the translation process [33]. Negative CIS elements depict the sequence motifs that negatively regulate gene expression. The value of negative CIS elements after codon optimization was successfully reduced from five to one [34].

The synthetic gene of interest was harbored in the plasmid pD451-SR, named pD451-SR-Pfupol, and transformed into *E.coli* BL21 Star (DE3) as the expression host. The gene was designed following the original sequence of the DNA polymerase gene from the hyper-thermophilic archaeon *Pyrococcus furiosus* with codon optimization. The transformant strain was able to grow in the medium containing kanamycin antibiotic because the resistance gene against kanamycin was present in its DNA plasmid. Based on the previous finding, the growth of the transformant strain was affected by the concentration of kanamycin. As engineered plasmids are likely lost during cultivation, the presence of a selective pressure such as antibiotics in the media is important to ensure plasmids’ stability [35, 36]. Once the transformation is performed, colony selection of transformants should be a pivotal step to do afterward. The transformant TC-02 with a high level of *Pfu* pol expression was opted as the parental colony, being employed to produce recombinant *Pfu* pol in an IPTG-induced LB medium.

There are several reasons for employing *E. coli* as the expression host. Its use as a cell factory is well-established and suitable for expressing stably folded proteins from prokaryotes and eukaryotes, and it has fast growth kinetics with a doubling time of about 20 min. Most importantly, our target protein has molecular weights of around 90 kDa which is very large and difficult to express in other hosts [37]. In *E. coli*, however, the target protein can be expressed in a soluble form.

To verify that a high level of the recombinant *Pfu* pol could be successfully overexpressed by the transformant colony with the induction of IPTG, the total protein from non-transformant and transformant colonies was compared. As shown in Fig. 3, a high level of *Pfu* pol expression can be found in the soluble fraction of a transformant. In contrast, no *Pfu* pol expression can be observed in a total fraction of a non-transformant colony. It indicates that the presence of a gene encoding *Pfu* pol in the transformant genome leads the transformant to be capable of producing recombinant *Pfu* pol and the induction of IPTG allows the transformant to express the high level of protein. Other researchers have also opted for a T7-based promoter system similar to our study [38, 39]. The advantage of using the system is that a T7-based promoter induces the enzyme to produce a more active and stable structure. This stability may help its structure intact during purification [40].

According to the similarities of primary amino acid sequence, DNA polymerases are classified into seven families; there are families A, B, C, D, E, X, and Y [41]. Taq DNA polymerase belongs to a Family A, including *Tth* and *Tma* DNA polymerases. They have 5′ to 3′ polymerase and 5’ to 3’ exonuclease activity but lack 3′ to 5′ exonuclease (proofreading) activity. Family A DNA polymerases have strong extension ability and high efficiency of amplification yet low fidelity. In the absence of a 3′ to 5′ exonuclease domain, the family A polymerases are prone to error while combining base pairs during DNA amplification. In contrast, *Pfu* DNA polymerase has been included in family B, along with *Kod* and *Tli* DNA polymerases. They possess intrinsic 3′ to 5′ exonuclease activity and are considered high fidelity [42]. However, the lack of 5′ to 3′ exonuclease activity causes their extension rate to be slow. Therefore, the idea of the PCR technique comes up with the combination of both DNA polymerases from Family A and Family B used to take advantage of both sides.

The purity of recombinant *Pfu* pol was obtained after performing the two-step purification. The crude enzyme was purified using a HisTrap™ HP column and followed by loading into a HiTrap™ Q HP column. The purification process using two chromatographic columns resulted in producing the purified *Pfu* pol with a predicted molecular weight of around 90 kDa. Various purification systems have developed to separate DNA polymerase from expression cells. An earlier study related to *Pfu* pol purification using chromatography has been carried out previously [43]. On the other hand, other investigators purified the polymerase enzyme using a simple method such as heat-based purification. The step was performed by heating the crude protein at 95 °C for around 5 min. As a result, most of the bacterial host proteins were denatured and only retained the thermostable *Pfu* pol [44, 45]. In addition, the purification of *Pfu* DNA polymerase can be completely performed by combining a heat treatment and the followed by several chromatographic processes [36, 46, 47].

The success of the purification process was then verified using western blot analysis. Our study confirmed that the identity of purified protein is certainly *Pfu* pol because the molecular weight of a single band obtained remains around 90 kDa. The result corresponded to the molecular weight predicted from the amino-acid sequence (775 aa). In accordance with our findings, some publications have reported the expression of *Pfu* pol using various host systems gave a similar molecular weight of 90 kDa, proving that the expression and DNA polymerases are not significantly affected by bacterial expression hosts [44, 48].

Subsequently, the specific activity of recombinant *Pfu* pol was measured using a qPCR system, and the functionality of the purified *Pfu* pol was tested using the standard PCR assay. Overall, the activity of the recombinant *Pfu* pol could be quantitatively assessed using the fluorometric method. The increased pattern in specific activity of the purified *Pfu* pol is proportional to the purification fold. The increment of purification was observed starting from 1.0-fold to 6.5-fold purification. According to our findings, we obtained 26.8 mg total purified protein with 292,820 U total activity in a 1-L culture. The amount of the purified protein obtained from a 1-L culture was higher than earlier studies reported by Zheng et al. (4.98 and 14.94 mg), Lu and Erickson (3.7 mg), and Sun and Cai (17 mg) which were obtained using non-optimized codon [11, 49, 50]. The result exhibited from our study indicated that the codon-optimized gene could improve the protein yield.

Furthermore, the value of the total activity of purified *Pfu* pol obtained in this study was twofold higher than that for *Pfu* pol purified with cellulose phosphate (P11) by Lu and Erickson (147,000 U) and was comparable for *Pfu* pol purified using JK110 chromatography (299,600 U) in a 1-L culture [11, 50]. The standard PCR assay of purified *Pfu* pol confirmed the functionality of the protein. The purified enzyme was fully functional to amplify the specific gene, generating a single band with roughly 900 base pairs in length. Around 1.0 and 1.25 units of purified *Pfu* pol were also applied for PCR assay and both samples gave a similar efficiency for DNA amplification.

## Conclusion

The recombinant *Pfu* DNA polymerase from a codon-optimized gene was successfully expressed in *Escherichia coli* BL21 Star (DE3) and purified using a two-step purification. The purified *Pfu* pol was achieved and confirmed using Western blot analysis with a molecular weight of approximately 90 kDa. According to our findings, *Pfu* pol showed its functionality and efficiency for DNA amplification. A high-level expression of *Pfu* pol of about 26.8 mg/L was achieved, indicating that our approach was suitable to be applied for the production of the recombinant *Pfu* pol.

## Data Availability

All data generated or analyzed during this study are included in this article.
